# Using the Risk Factors of Pancreatic Cancer and Their Interactions in Cancer Screening: A Case-Control Study in Shanghai, China

**DOI:** 10.5334/aogh.2463

**Published:** 2019-07-11

**Authors:** Xiaojie Bo, Jianwei Shi, Rui Liu, Shasha Geng, Qingqing Li, Yang Li, Hua Jin, Sen Yang, Hua Jiang, Zhaoxin Wang

**Affiliations:** 1 Department of Pediatrics, Shanghai Tenth People’s Hospital, Tongji University School of Medicine, Shanghai, CN; 2 School of Public Health, Shanghai Jiaotong University School of Medicine, Shanghai, CN; 3 Department of General Practice, Yangpu Hospital, Tongji University School of Medicine, Shanghai, CN; 4 Shanghai East Hospital, Tongji University School of Medicine, Shanghai, CN; 5 Academic Department of General Practice, Tongji University School of Medicine, Shanghai, CN; 6 Shanghai General Practice and Community Health Development Research Center, Shanghai, CN

## Abstract

**Background:**

The incidence of pancreatic cancer has increased annually, but the risk factors and their interactions are still unknown.

**Objective:**

The aim of this study was to identify risk factors and the effects of their interactions on pancreatic cancer occurrence among patients in Shanghai, China.

**Methods:**

We conducted a hospital-based case-control study. The case group consisted of pathologically diagnosed pancreatic cancer patients, and the control group consisted of a healthy population. The Pearson Chi-square test was used to compare the distribution frequencies of data between groups. Multivariate analysis and interaction analysis were conducted to explore possible risk factors and interactions between various variables.

**Findings:**

Among the 4,821 recruited participants, 1,392 were pancreatic cancer patients and 3,429 were controls. Multivariate logistic analysis suggested that age (>50 years old) (AOR: 16.20 [95% CI 6.78; 38.69]), diabetes (AOR: 5.40 [95% CI 2.70; 10.80]), chronic pancreatitis (AOR: 27.43 [95% CI 2.14; 351.77]), smoking (AOR: 8.86 [95% CI 3.07; 25.58]), and family cancer history (AOR: 2.10 [95% CI 1.09; 8.56]) were the primary risk factors for pancreatic cancer. Interestingly, synergistic interactions between risk factors were found, especially between age and chronic pancreatitis (RERI = 447.93, API = 96.74%, SI = 32.78), age and smoking (RERI = 187.42, API = 94.97%, SI = 21.99), and diabetes and smoking (RERI = 14.39, API = 48.06%, SI = 1.99).

**Conclusions:**

Age, diabetes, chronic pancreatitis, smoking, and family cancer history have been verified as the primary risk factors for pancreatic cancer in this study. Moreover, the interaction effects between old age, diabetes, chronic pancreatitis, and smoking substantially increase the probability of the development of pancreatic cancer. Cancer screening should be conducted extensively among people with these multiple factors to improve the efficiency.

## Strengths and limitations of this study are as follows

1. This study confirmed the significance of previously established risk factors for pancreatic cancer.
2. This study found significant synergistic interactions between some risk factors for pancreatic cancer.
3. The results of this study contribute to the early prevention and screening of pancreatic cancer.
4. As a hospital-based case-control study, selection bias was inevitable.
5. The study period was limited by relevant information on body mass index and diet, and detailed information on the amount of smoking and alcohol consumption was not available.

## Introduction

Pancreatic cancer is a highly lethal disease, with a mortality rate that is very close to its incidence rate [1]. With no specific observable symptoms until in its advanced and incurable stage, pancreatic cancer develops insidiously [2] Due to the late diagnosis, patients with pancreatic cancer have one of the worst five-year survival rates of all cancers (6% in the US) [1]. Currently, the number of deaths from pancreatic cancer is increasing, and it is predicted to be the second-leading cause of cancer-related death in the US by 2030 [3]. Similarly, an increase in pancreatic cancer mortality has been reported in European populations [4]. In China, the incidence and mortality rates of pancreatic cancer increased steadily from 2000 to 2011. In 2015, the number of male and female pancreatic cancer patients in China reached 52,200 and 37,900, respectively, and the numbers of deaths were 45,600 and 33,800, respectively [5].

Similar to most malignancies, pancreatic cancer results from the combination of environmental factors and genetic factors [6]. Although studies have reached a consensus on the etiology of pancreatic cancer, the epidemiological causes of pancreatic cancer identified in different studies have varied, and the influence of these factors on pancreatic cancer is still under debate [7]. At present, smoking has been identified as the most well-established risk factor for pancreatic cancer on a global scale [8,9,10]. Other factors, such as age, diabetes and chronic pancreatitis, have also been associated with pancreatic cancer in most recent studies [11,12]. Possible interactions between the risk factors may exist due to the multifactorial nature of pancreatic carcinogenesis. However, few studies have examined the possible interactions, and no large case-control studies have been conducted on the interactions among the risk factors for pancreatic cancer.

Considering that there are few studies on the interactions between various risk factors for pancreatic cancer in China, we conducted this study to identify these risk factors and their interactions, aiming to contribute to the early prevention of and screening for pancreatic cancer.

## Methods

### Study design

A hospital-based case-control study was conducted to identify the risk factors for pancreatic cancer and their interactions based on data from pancreatic cancer patients from 14 tertiary hospitals in Shanghai, China. A total of 4,821 participants were recruited, including 1,392 with pathologically verified cancer and 3,429 healthy controls. All participants were selected from fourteen tertiary hospitals in Shanghai.

All of the primary pancreatic cancer patients were diagnosed in the oncology departments of the above fourteen tertiary hospitals between January 1, 2012, and December 12, 2017. The inclusion criteria for the case group were as follows: pathologically diagnosed pancreatic ductal adenocarcinoma and literate. The exclusion criteria were as follows: patients diagnosed with other types of pancreatic disease, such as neuroendocrine tumor, adenomas, cysts, or other unknown primary tumors; concurrent cancer at another organ site; and a history of other cancers.

Participants in the control group were recruited from the physical examination center of the same hospitals; their physical medical examination results indicated that they were healthy. According to the ages recorded on the physical examination form, we chose the control group such that their age composition matched that of the total population of Shanghai. Moreover, controls and cases were matched according to sex and race. The eligibility criteria for the controls were the same as those for the patients, except for the cancer diagnosis.

### Study participants

In this study, a total of 1,903 pancreatic cancer cases were selected through the hospital registry system once they were diagnosed with pancreatic cancer. For the controls, 4,848 were chosen when they received a health examination. Then, each participant was asked to provide informed consent to participate in a structured questionnaire. Some of the questionnaires were finished when the participants were in the hospitals, and the rest were finished over the telephone. All the questionnaires were administered by our trained investigators. Finally, we checked the information pertaining to medical history on the questionnaire by comparing it with the participant’s medical file and then excluded the participants with inconsistent information. In total, the participation rates of patients in the case group and the control group were 73.15% (1392/1903) and 70.73% (3429/4848), respectively. The loss was due to the following reasons: inconsistent information and refusal to answer the questionnaire. A total of 4,821 (1,392 cases, 3,429 controls) participants were enrolled, and there were no significant differences between the case group and the control group in terms of the characteristics of gender, race, marital status, and so on.

### Questionnaire data

The questionnaire was designed by relevant experts from the hospital and university who have abundant experience in clinical diagnosis or screening for pancreatic cancer. The hospital staff members were available for assistance if participants had enquiries regarding the questions. The questionnaire was divided into three sections: section 1 contained questions on demographic characteristics, such as age, gender, race and marital status; section 2 contained questions on smoking status and alcohol consumption; and section 3 was about the participant’s medical history and family cancer history. All information was collected by self-report. All questionnaires were cross-checked daily by the investigators.

### Data analysis

All analyses were performed with SPSS software (version 21.0; SPSS, Inc., Chicago, IL, USA). Chi-square tests were used to assess the differences between the case and control groups. First, univariate analysis was conducted for each relevant risk factor. Based on univariate analysis of the relevant risk factors combined with literature reports and clinical experience, all the risk factors with screening value were then included in multiple logistic regression models to evaluate their independent effects. Multivariate analysis was adjusted for potential confounders, such as sex, race, and marital status, to evaluate the association of exposure risk factors with the risk of pancreatic cancer. The adjusted odds ratios (AORs) and 95% confidence intervals (CIs) of the associations between the risk factors and pancreatic cancer risk were calculated by multivariate logistic analysis.

Moreover, we used multiple logistic regression models to investigate possible interactions between the risk factors. We chose to use an additive model rather than a multiplicative model because the former has a more appropriate scale for addressing biologic interactions and public health concerns. To assess deviation from the additive model (which assumes there is no interaction between variables), the relative excess rate due to interaction (RERI = OR_11_ – OR_01_ – OR_10_ + 1), the attributable proportion due to interaction (API = (RERI/OR_11_) and the synergism index (SI = [OR_11_ – 1]/([OR_01_ + OR_10_] – 2) were calculated, where OR_11_ = the OR of the joint effect of two risk factors, and OR_10_ and OR_01_ = the OR of each risk factor in the absence of the other [13].

## Results

### Participant demographics

This hospital-based case-control study included 1,392 cases of pancreatic cancer patients and 3,429 healthy cases. Table 1 summarizes the demographic characteristics of our sample in both the case and control groups. The ratio of male to female was slightly higher than 1:1, and there was no significant difference in the sex ratio between the case and control groups. All patients in the case group and the vast majority in the control group (97.75%) were of Han ethnicity. There was no significant difference in the sex ratio or the race ratio between the cases and controls. Concerning marital status, most of the participants in the case (99.43%) and control groups (82.62%) were married, but there was still a significant difference between the two groups (P < 0.001). We chose to adjust for all demographic factors in the multivariate logistic regression models.

**Table 1:** Characteristics of the study participants.

Study Variables	Patients (N = 1,392)		Controls (N = 3,429)		χ^2^	*P*	OR(95% CI)

	n	%	n	%			

Sex							
Male	830	59.63	1,981	57.77	0.16	0.689	/
Female	562	40.37	1,448	42.23			
Race							
Han ethnicity	1,392	100.00	3,352	97.75	3.29	0.069	/
Others	0	0.00	77	2.25			
Marital status							
Married	1,384	99.43	2,833	82.62	25.18	0.000	/
Unmarried	8	0.57	596	17.38			
Age (years)							
<50	71	5.10	1,507	43.95			1.00
50–59	249	17.89	721	21.03	21.87	0.000	25.00 (3.41–200.00)
60–69	501	35.99	452	13.18	63.18	0.000	76.92 (10.20–500.00)
70–79	313	22.49	228	6.65	91.48	0.000	111.11 (1.52–1000.00)
≥80	258	18.53	521	15.19	108.51	0.000	142.86 (19.23–1000.00)
Hypertension							
Yes	660	47.41	1,038	30.27	12.76	0.000	2.08 (1.39–3.11)
No	732	52.59	2,391	69.73			1.00
Hyperlipidemia							
Yes	23	1.65	671	19.57	26.41	0.000	0.06 (0.02–0.25)
No	1.369	98.35	2,758	80.43			1.00
Diabetes							
Yes	511	36.71	359	10.47	45.89	0.000	4.94 (3.03–8.05)
No	881	63.29	3,070	89.53			1.00
Chronic pancreatitis							
Yes	38	2.73	14	0.41	50.01	0.001	6.85 (3.70–12.68)
No	1,354	97.27	3,415	99.59			1.00
Biliary disease							
Yes	391	28.09	622	18.14	5.95	0.015	1.77 (1.12–2.80)
No	1.001	71.91	2,807	81.86			1.00
History of surgical trauma							
Yes	803	57.69	1,371	39.98	12.39	0.000	2.04 (1.37–3.04)
No	589	42.31	2,058	60.02			1.00
Smoking							
Yes	232	16.67	112	3.27	268.31	0.000	5.92 (4.68–7.49)
No	1,160	83.33	3,317	96.73			1.00
Family cancer history							
Yes	94	6.75	124	3.62	22.56	0.000	1.93 (1.47–2.54)
No	1,298	93.25	3,305	96.38			1.00

### Univariate analysis

Table 1 shows the results of the univariate analysis by Chi-square tests. According to the preliminary results, we found that there were no significant differences in the risk of pancreatic cancer among people aged 18–29 years old, 30–39 years old, and 40–49 years old; hence, they were combined in one group under 50 years old. The results show that increasing age can increase the risk of pancreatic cancer; with less than 50 years old as the reference, the odds ratios are as follows: from 50 to 59 years old (OR = 25.00, 95% CI [3.41–200.00]), 60 to 69 years old (OR = 76.92, 95% CI [10.20–500.00]), 70 to 79 years old (OR = 111.11, 95% CI [1.52–1000.00]), and 80 years old or older (OR = 142.86, 95% CI [19.23–1000.00]). The risk of pancreatic cancer increases gradually as age increases. The results also suggest that other possible risk factors include hypertension (OR = 2.08, 95% CI [1.39–3.11]), diabetes (OR = 4.94, 95% CI [3.03–8.05]), chronic pancreatitis (OR = 6.85, 95% CI [3.70–12.68]), biliary disease (OR = 1.77, 95% CI [1.12–2.80]), a history of surgery or trauma (OR = 2.04, 95% CI [1.37–3.04]), smoking (OR = 5.92, 95% CI [4.68–7.49]), and family cancer history (OR = 1.93, 95% CI [1.47–2.54]). In contrast, hyperlipidemia (OR = 0.06, 95% CI [0.02–0.25]), is a protective factor. However, alcohol consumption (*P* = 0.721) was not statistically significant in the univariate analysis.

### Multivariate analysis

To assess the independent effects of different risk factors on the incidence of pancreatic cancer and to control for interference from potential confounding factors, we conducted a multivariate analysis using a logistic regression model. Based on the results of the univariate analysis, we divided the participants into two age groups, with the cutoff value of 50 years old. We incorporated all the potential confounding factors and the risk factors into a multiple regression model, and the final model equation contained a total of five variables (Table 2). The results showed that people with diabetes (AOR = 5.40, 95% CI [2.70–10.80]), chronic pancreatitis (AOR = 27.43, 95% CI [2.14–351.77]), old age (AOR = 16.20, 95% CI [6.78–38.69]), smoking (AOR = 8.86, 95% CI [3.07–25.58]), and a family cancer history (AOR = 2.1, 95% CI [1.09–8.56]) had a higher risk. All of the results were obtained after adjusting for sex, race, marital status, and other risk factors.

**Table 2:** Risk factors for pancreatic cancer in multivariable logistic regression analysis.

Variables	β	SE (β)	Wald (*χ*^2^)	AOR (95% CI)	P

Diabetes					
Yes	1.69	0.35	22.73	5.40 (2.70–10.80)	0.000
No				1.00	
Chronic pancreatitis					
Yes	3.31	1.3	6.47	27.43 (2.14–351.77)	0.011
No				1.00	
Smoking					
Yes	2.18	0.54	16.24	8.86 (3.07–25.58)	0.000
No				1.00	
Family cancer history					
Yes	1.14	0.23	20.21	2.1 (1.09–8.56)	0.000
No				1.00	
Age, years					
<50	2.79	0.44	39.3	16.20 (6.78–38.69)	0.000
≥50				1.00	

Adjusted for gender, race, marital status, hypertension, hyperlipidemia, biliary disease, history of surgical trauma, and drinking.

### Interaction analysis

For the above four primary risk factors that were statistically significant in the multivariate logistic regression model with an AOR value larger than 1, an interaction analysis was further performed with the logistic regression model and the additive model.

Table 3 shows the independent and interactive effects of age, diabetes, chronic pancreatitis, and smoking on pancreatic cancer incidence. The results indicated additivity and synergism between age and chronic pancreatitis, age and smoking, and diabetes and smoking, after adjusting for the effects of other significant risk factors.

**Table 3:** Synergistic interactions among age, diabetes, chronic pancreatitis and smoking.

Interaction Variables		AOR (95% CI)	RERI	API	SI

Variable 1	Variable 2				

**Age^1^**	**Diabetes^1^**				
≤50 years	No	1	–31.43	–26.38%	0.79
≤50 years	Yes	123.06 (10.22–1482.18)			
>50 years	No	28.50 (9.38–86.62)			
>50 years	Yes	119.14 (33.91–418.55)			
**Age^2^**	**Chronic pancreatitis^2^**				
≤50 years	No	1	447.93	96.74%	32.78
≤50 years	Yes	/			
>50 years	No	16.10 (6.73–38.48)			
>50 years	Yes	463.03 (29.54–7258.03)			
**Age^3^**	**Smoking^3^**				
≤50 years	No	1	187.42	94.97%	21.99
≤50 years	Yes	/			
>50 years	No	10.93 (4.66–25.64)			
>50 years	Yes	197.35 (40.49–962.03)			
**Diabetes^4^**	**Chronic pancreatitis^4^**				
No	No	1	/	/	/
No	Yes	23.25 (1.58–342.12)			
Yes	No	5.35 (2.67–10.71)			
Yes	Yes	/			
**Diabetes^5^**	**Smoking^5^**				
No	No	1	14.39	48.06%	1.99
No	Yes	10.48 (3.38–32.52)			
Yes	No	6.07 (2.85–12.91)			
Yes	Yes	29.94 (5.36–167.33)			
**Chronic pancreatitis^6^**	**Smoking^6^**				
No	No	1	/	/	/
No	Yes	8.83(3.05–25.55)			
Yes	No	26.21 (1.76–389.40)			
Yes	Yes	/			

^1^ Adjusted for sex, race, marital status, hypertension, hyperlipidemia, biliary disease, history of surgical trauma, alcohol consumption, chronic pancreatitis, and smoking. ^2^ Adjusted for sex, race, marital status, hypertension, hyperlipidemia, biliary disease, history of surgical trauma, alcohol consumption, diabetes, and smoking. ^3^ Adjusted for sex, race, marital status, hypertension, hyperlipidemia, biliary disease, history of surgical trauma, alcohol consumption, diabetes, and chronic pancreatitis. ^4^ Adjusted for age, sex, race, marital status, hypertension, hyperlipidemia, biliary disease, history of surgical trauma, alcohol consumption, and smoking. ^5^ Adjusted for age, sex, race, marital status, hypertension, hyperlipidemia, biliary disease, history of surgical trauma, alcohol consumption, and chronic pancreatitis. ^6^ Adjusted for age, sex, race, marital status, hypertension, hyperlipidemia, biliary disease, history of surgical trauma, alcohol consumption, and diabetes. /: P > 0.05.

The RERIs were 447.93, 187.42, and 14.39 for the age/chronic pancreatitis, age/smoking, and diabetes/smoking interactions, respectively. Using the RERIs, we estimated that the attributable proportion due to interaction (API %) values explained by age/chronic pancreatitis, age/smoking, and diabetes/smoking interaction were 96.74%, 94.97%, and 48.06%, respectively. The estimated SI values were 32.78, 21.99, and 1.99, respectively. This may indicate that the interaction effect of the above three groups of pancreatic cancer risk factors is super additive. Additionally, the results revealed that there was a negative interaction between age and diabetes (RERI = –31.43, API = –26.38%, SI = 0.79). No other significant interaction was observed between groups, such as diabetes and chronic pancreatitis and chronic pancreatitis and smoking.

## Discussion

Studies have suggested that the development of pancreatic cancer is a complex and multistep process with a multifactorial etiology, as many factors (genetic susceptibility, environmental factors, lifestyles, and physical conditions) may contribute to its occurrence [14,15,16]. In our study, we found that pancreatic cancer is more common among older adults, and there was a significantly higher risk associated with older age. Consistent with our results, Qi Dong et al. retrospectively analyzed the clinical data of 207 patients with pancreatic cancer, and their study showed that age is an independent risk factor for the incidence of pancreatic cancer. The relative risk of pancreatic cancer increased approximately 1.78 times in the older group compared with the younger group [17]. One possible reason may be that aging causes the body to accumulate more carcinogens and changes some of the body’s defense mechanisms [18]. This can be one of the pathophysiological mechanisms underlying the development of pancreatic cancer. It is predicted that the incidence of pancreatic cancer will continue to rise as the aging of the population continues to accelerate.

Diabetes can be a cause and a complication of pancreatic cancer, although reports are not consistent. Our results indicated that diabetes is an important risk factor for pancreatic cancer. This is in accordance with the results of the meta-analysis conducted by Huxley et al., which analyzed 19 cohort studies and 17 case-control studies between 1966 and 2005 [18]. Zhan et al. conducted a retrospective study of 2,405 patients with malignant tumors and found that 28% of the patients had been diagnosed with diabetes before developing cancer. Among them, the percentage of pancreatic cancer patients (57.1%) was the highest, which suggested that diabetes may be a risk factor for pancreatic cancer, and it is advisable to perform conventional screening of diabetic patients for the early diagnosis of pancreatic cancer [19]. However, according to Liao et al.’s 10-year population-based cohort study (49,803 vs. 199,212) based on the Taiwan National Health Insurance Database, diabetes with <2 years’ duration was associated with pancreatic cancer and could be an early manifestation of pancreatic cancer, but long-standing diabetes was not found to be a risk factor for pancreatic cancer [20]. However, we did not obtain information on the duration of diabetes in this study. Therefore, the association of diabetes with pancreatic cancer warrants further investigation.

Some studies have concluded that various types of chronic pancreatitis, including alcoholic, non-alcoholic, hereditary, and tropical pancreatitis, are associated with pancreatic cancer to some extent [21]. A significant association between chronic pancreatitis and pancreatic cancer risk was found in our study. The results of our multivariate logistic analysis showed that the risk for pancreatic cancer was 20-fold higher among patients affected by chronic pancreatitis after adjusting for potential confounders. In previous studies, most authors had drawn similar conclusions, and the relative risks for pancreatic cancer in chronic pancreatitis patients varied from 2.3 to 18.5 [22]. Furthermore, a recent meta-analysis conducted by Raimondi et al. of 22 studies that found that the risk of developing pancreatic cancer in patients with chronic pancreatitis was 20 times higher than among those without chronic pancreatitis [23]. In addition, chromosomal instability in patients with chronic pancreatitis is also conducive to the occurrence of pancreatic cancer. Therefore, we believe that patients with chronic pancreatitis should be considered at high risk for pancreatic cancer, and they would benefit from preventive measures and early detection programs.

According to other studies, smoking has been definitively identified as the most important environmental risk factor for pancreatic cancer [24]. To date, at least 30 epidemiological studies have confirmed this view [8,9,25,26,27,28]. The results of our multivariate analysis also revealed that smoking was associated with an increased risk of pancreatic cancer. Consistent with our results, a nested case-control study conducted by Lynch S.M. et al., including 1,481 cases and 1,539 controls, found that the ORs of pancreatic cancer were 1.1 (95% CI, 0.9–1.3) for former smokers and 1.8 (95% CI, 1.4–2.3) for current smokers compared to nonsmokers [29]. Similar findings were obtained in a large hospital-based case-control study (808 vs. 808) and a 40-year follow-up cohort study [30,31]. According to pathological studies, the effect mechanism may result from the carcinogens in tobacco acting on the pancreas through the blood or duodenal fluid and bile reflux [32].

Consistent with our results, Schulte’s study, which included 591 pancreatic cancer patients and 646 controls, reported that a family history of pancreatic cancer (OR 2.20, 95% CI 1.16–4.19) and melanoma (OR 1.74, 95% CI 1.03–2.95) were risk factors for pancreatic cancer [30]. Hassan’s case-control study showed that family histories of cancer in general and of pancreatic cancer in particular were associated with a significantly elevated risk for pancreatic cancer. The effect of a positive family history of pancreatic cancer was significant in women and men. The pattern was consistent with the familial predisposition reported for pancreatic cancer and with the array of tumors associated with hereditary nonpolyposis colon cancer. In our analysis, hyperlipidemia had a negative association with pancreatic cancer. The results are inconsistent with other studies showing that high blood lipid levels increase the risk of pancreatic cancer [33], and possible reasons were related to the use of statins for treating hyperlipidemia. Regular statin use before the diagnosis of pancreatic cancer was associated with modest increases in survival times in two large prospective cohort studies [34]. In our study, it is possible that the patients did not take statins to treat hyperlipidemia. However, we did not obtain this information from the questionnaires, and the association needs to be explored in future analyses.

The most noteworthy finding of our study was the interactions between certain risk factors. Our results showed that there were significant positive synergistic interactions between age and chronic pancreatitis, age and smoking, and diabetes and smoking after adjusting for the effects of other significant risk factors. This supports the hypothesis that people with a combination of two or more risk factors are more vulnerable to pancreatic cancer. Similar to our research design, a large case-control study (808 vs. 808) conducted by Hassan et al. found that synergistic interactions did exist between cigarette smoking and a family history of pancreatic cancer (AOR = 12.8, 95% CI 1.6–108.9) and between smoking and diabetes (AOR = 9.3, 95% CI 2.0–44.1) in women. Moreover, a recent study in Italy in 2014 found that important synergistic interactions existed between smoking and alcohol consumption (SI = 17.61) and alcohol consumption and diabetes (SI = 17.77) [35]. However, in our study, similar to in other studies, we did not find a significant interaction between chronic pancreatitis and smoking, and a possible mechanism still needs to be explored. To a certain extent, this study provided insight into whether the performance of pancreatic cancer screening among people with multiple risk factors, such as old age, diabetes, and smoking, would substantially improve the efficiency of cancer screening.

The limitations of the study should be taken into account. First, our study is a case-control study that applies an analytical epidemiological research method that has inherent limitations and disadvantages. The selection bias and recall bias cannot be eliminated completely. To address the selection bias problem, we adopted a multicenter design in which the cases and controls were recruited from the same hospitals. We selected newly diagnosed cases and avoided using proxy respondents, minimizing recall bias. Second, we did not obtain relevant information on body mass index, diet, details regarding smoking and alcohol consumption, or the duration of diabetes. Therefore, further investigations into pancreatic cancer risk factors are warranted.

## Conclusion

The results of this study show that age, diabetes, chronic pancreatitis, smoking, and family cancer history are risk factors for pancreatic cancer. Moreover, the interactions of the risk factors increase the probability of developing pancreatic cancer. This study provides important clues for the prevention of pancreatic cancer. We believe that identifying individuals with the highest risk of pancreatic cancer can contribute greatly to the development of prevention programs and the early detection of pancreatic cancer. More large-scale epidemiological studies with different populations on the interactions among risk factors are warranted, with stratification by sex.

## Abbreviations

AOR: Adjusted Odd Ratio

CI: Confidence Interval

RERI: Relative Excess Rate due to Interaction

API: Attributable Proportion due to Interaction

SI: Synergism Index

## Author Information

Xiaojie Bo and Jianwei Shi are co-first authors in this article.
